# Telomeres and aging: *on and off the planet!*

**DOI:** 10.1007/s10522-024-10098-7

**Published:** 2024-04-06

**Authors:** Christopher E. Mason, Maria A. Sierra, Henry J. Feng, Susan M. Bailey

**Affiliations:** 1 Department of Physiology and Biophysics, Weill Cornell Medicine, New York, NY USA; 2 The HRH Prince Alwaleed Bin Talal Bin Abdulaziz Alsaud Institute for Computational Biomedicine and WorldQuant Initiative for Quantitative Prediction, Weill Cornell Medicine, New York, NY USA; 3 Tri-Institutional Computational Biology & Medicine Program, Weill Cornell Medicine, New York, NY USA; 4 Department of Biological Sciences, Columbia University, New York, NY USA; 5 Present Address: Faculty of Medicine and Health, Sydney Medical School, University of Sydney, Sydney, Australia; 6 Department of Environmental and Radiological Health Sciences, Colorado State University, Fort Collins, CO USA

**Keywords:** Telomeres, Aging, Space, Radiation, Omics, Oxidative stress, Mitochondrial dysregulation

## Abstract

Improving human healthspan in our rapidly aging population has never been more imperative. Telomeres, protective “caps” at the ends of linear chromosomes, are essential for maintaining genome stability of eukaryotic genomes. Due to their physical location and the “end-replication problem” first envisioned by Dr. Alexey Olovnikov, telomeres shorten with cell division, the implications of which are remarkably profound. Telomeres are hallmarks and molecular drivers of aging, as well as fundamental integrating components of the cumulative effects of genetic, lifestyle, and environmental factors that erode telomere length over time. Ongoing telomere attrition and the resulting limit to replicative potential imposed by cellular senescence serves a powerful tumor suppressor function, and also underlies aging and a spectrum of age-related degenerative pathologies, including reduced fertility, dementias, cardiovascular disease and cancer. However, very little data exists regarding the extraordinary stressors and exposures associated with long-duration space exploration and eventual habitation of other planets, nor how such missions will influence telomeres, reproduction, health, disease risk, and aging. Here, we briefly review our current understanding, which has advanced significantly in recent years as a result of the NASA Twins Study, the most comprehensive evaluation of human health effects associated with spaceflight ever conducted. Thus, the Twins Study is at the forefront of personalized space medicine approaches for astronauts and sets the stage for subsequent missions. We also extrapolate from current understanding to future missions, highlighting potential biological and biochemical strategies that may enable human survival, and consider the prospect of longevity in the extreme environment of space.

## Main text

### Telomeres and telomerase: essential to human health

Telomeres*,* nucleoprotein complexes that “cap” the ends of linear chromosomes, are composed of highly conserved, tandem arrays of G-rich repetitive sequence (5′-TTAGGG-3′ in humans, vertebrates) (Moyzis et al. 1988; Meyne et al. 1989). Telomeres terminate in a G-rich 3′ single-stranded (ss) overhang, and both double-stranded (ds) and ss-telomeric regions are bound by an assortment of proteins, collectively termed shelterin, which serve to protect chromosomal termini from degradation and fusion (de Lange 2005). Telomeres also prevent the natural ends of chromosomes from being mistakenly recognized as broken DNA [double-strand breaks (DSBs)] and triggering deleterious DNA damage responses (DDRs; de Lange 2002). Due to the end-replication problem first envisioned by Olovnikov (1971), and later by Watson (1972), telomere length naturally erodes with cell division and thus with aging, causing telomeres to shorten until reaching a critically shortened length, at which point their ability to provide end-protection and prevent DDRs is lost, and a permanent cell cycle arrest known as replicative senescence is entered (Hayflick 1965; Harley et al. 1990). In cells with defective checkpoint machinery (including most cancer cells), DDRs that mis-repair dysfunctional telomeres and/or broken DNA ends can result in telomere fusions, chromosome rearrangements, and rampant genome instability, known drivers of tumorigenesis (Chin et al. 1999). Loss of telomere function can result either from alterations that promote the gradual or sudden loss of sufficient repeat sequence necessary to maintain proper telomere structure, or from deficiencies in the telomere-associated proteins required for end-capping function (Bailey and Murnane 2006; Bailey et al. 1999).

### The telomere paradox

In normal cells, dysfunctional telomere-induced senescence serves as an effective barrier to unlimited cell growth or replicative immortality, a key hallmark of cancer, and therefore represents an important tumor suppressor mechanism (Maciejowski and de Lange 2017; Hanahan 2022). However, senescence also underlies significant phenotypes associated with aging; e.g., the senescence-associated secretory phenotype (SASP) promotes chronic inflammation (inflammaging) and drives degenerative pathologies and carcinogenesis (Campisi 2001, 2013; Campisi et al. 2001; Coppe et al. 2008). Discovery of SASP prompted development of a new class of small molecules termed senolytics that selectively target and kill senescent cells as a means of delaying or preventing age-related diseases (Zhu et al. 2015), and have shown promise and efficacy in humans (Ellison-Hughes 2020). Telomere shortening also triggers global reductions in histone levels and epigenetic changes (O'Sullivan et al. 2010), alterations that have been implicated in aging and age-related disease; e.g., DNA methylation biological “clocks” (Salameh et al. 2020; Pearce et al. 2022). Emerging evidence suggests intriguing interplay between telomere attrition and metabolic compromise/imbalance or mitochondrial dysregulation/damage in senescence and the aging process, as well (Gao et al. 2022). Such findings are consistent with a previously reported positive association between mtDNA and telomere length in a community of healthy adults, which suggested co-regulation of telomeres and mitochondrial function (Tyrka et al. 2015).

Premature telomere attrition, as seen in individuals with variants in telomerase or shelterin proteins, causes dyskeratosis congenita and other diseases on a growing list of short telomere syndromes or telomeropathies, which share a common theme of bone marrow failure and cancer predisposition (Armanios and Blackburn 2012). Thus, telomere attrition acts as a double-edged sword, promoting tumor suppression while also limiting cell lifespan. This long-recognized paradox (Shay and Wright 2019; Maciejowski and de Lange 2017; Nassour et al. 2021) has recently been unified in the ‘Telomere Erosion in Disposable Soma’ theory of aging, which articulates the advantages of telomere loss-induced tumor suppression early in life, combined with compromised cell renewal and consequent increased disease risk later in life (Lansdorp 2022). Indeed, shortened telomere length has been linked to a spectrum of age-related degenerative pathologies, including reduced immune function, loss of fertility, dementias, cardiovascular disease (CVD), and cancer (Cohen et al. 2013; Honig et al. 2012; Haycock et al. 2014; Shay 2013a, 2013b).

### Telomerase functions to modulate telomere shortening

Precisely as Dr. Olovnikov originally theorized, the cellular mortality enforced by progressive telomere shortening can be circumvented via a “compensatory DNA polymerase”, the now well-recognized and specialized reverse transcriptase, Telomerase (TERT; catalytic subunit), whose integral RNA component (TERC) serves as a template for de novo addition of telomere repeats onto newly replicated chromosomal termini, thereby counteracting sequence loss due to the end-replication problem (Greider and Blackburn 1985; Falus et al. 2010). Confirmatory experimental evidence culminated in the demonstration that expression of TERT is sufficient to confer immortalization of human fibroblasts in culture (Bodnar et al. 1998). However, telomerase activity is repressed in most human somatic cells around the time of birth, so telomerase activity remains sufficient to maintain telomere length only in highly proliferative populations, including germline and stem cells, and the vast majority of cancer cells in which mutations in the *TERT* promoter region or alternative splicing of the TERT transcript endow them with unlimited replicative potential (Kim et al. 1994; Batista 2014; Dratwa et al. 2020; Penev et al. 2021; Cong et al. 2002). The remaining ~ 10–15% of human cancers lacking telomerase activity maintain telomere length via the recombination-mediated Alternative Lengthening of Telomeres (ALT) pathway (Bryan et al. 1997; Murnane et al. 1994), which is strongly associated with functional loss of the ATRX-DAXX chromatin-remodeling complex (Graham et al. 2019) and elevated levels of telomeric RNA, or TERRA (Flynn et al. 2015; Nguyen et al. 2017; Azzalin et al. 2007; Schoeftner and Blasco 2008). ALT cells present a variety of defining features including heterogeneous telomere lengths (very long and very short), increased frequencies of telomeric sister chromatid exchange (T-SCE), ALT-associated PML bodies (APBs), and extrachromosomal telomeric repeats (ECTRs) that include C-rich (ss)circles (Bailey et al. 2004; Henson et al. 2009; Cesare and Reddel 2010). The ALT phenotype is relatively common in several subtypes of human sarcomas, astrocytomas, and neuroblastomas, and has been observed in ~ 4% of all tumor types, including carcinomas and pediatric glioblastoma multiforme (Heaphy et al. 2011).

### Many factors influence telomere length

Interestingly, telomere length varies considerably even among same-aged individuals, with heterogeneity being present at birth and ranging from ~ 5 to 15 kb over the lifespan in humans (Samassekou et al. 2010; Martens et al. 2021). Telomere length, and more recently appreciated telomere sequence variants, are inherited traits with a higher concordance and rate of paternal transmission (Njajou et al. 2007; Grigorev et al. 2021). Moreover, telomere length diminishes not only with normal aging (Honig et al. 2015; Aubert and Lansdorp 2008), but also with oxidative stress and inflammation (von Zglinicki 2000; Zhang et al. 2016). Due to the G-rich nature of the telomere repeat sequence, telomeric regions are particularly susceptible to oxidative damage (e.g., reactive oxygen species; ROS), a consequence of normal cellular metabolism and exogenous exposures. Stressors, such as ionizing radiation, generate increased ROS production, thus telomeres, especially short telomeres, are extremely sensitive to radiation exposure, and so have been proposed as hallmarks of individual radiosensitivity and long-term or late effects of exposure (Ayouaz et al. 2008; Sridharan et al. 2015; Mirjolet et al. 2015). Furthermore, oxidative damage tends to accumulate because telomeres are also refractory to repair (Miller et al. 2011; Fumagalli et al. 2012), making them useful aggregate biomarkers of damage. Chronic oxidative stress has been shown to transiently activate the ALT pathway and/or induce ALT-like phenotypes (Coluzzi et al. 2017; Liu et al. 2018), including in vivo exposures associated with living the extreme environment of space (Luxton et al. 2020a, 2020b).

Telomere length is influenced by a variety of other factors as well, including biological sex (Gardner et al. 2013), lifestyle factors (e.g., diet Gu et al. 2015, smoking and obesity Valdes et al. 2005, physical activity Cherkas et al. 2008, psychological stress Epel et al. 2004, adverse childhood experiences (ACEs) Burgin et al. 2019, socioeconomic status (SES) Alexeeff et al. 2019, chronic stress and disease Blackburn and Epel 2012]. Telomeres can be regarded as informative sentinels of environmental exposures, such as air pollution, UV and ionizing radiations as well, since these also influence telomere length (Miri et al. 2019; Rochette and Brash 2010; Shim et al. 2014). Therefore, telomere length and sequence maintenance over time (telomere length dynamics) represents a key integrating component of the cumulative effects of genetics, lifestyle factors, and environmental exposures; i.e., it is the rate at which telomeres shorten—and not their absolute length—that provides a robust biomarker, and even determinant of general health and disease risk, and thus correlates with lifespan (Whittemore et al. 2019).

We and others have shown that longitudinal analyses of telomere length within individuals is more informative than cross-sectional comparison at single time points (across individuals); an illustrative example involved predicting potential risk of degenerative effects following radiation therapy in prostate cancer patients undergoing IMRT (Luxton et al. 2021), which was consistent with data that telomere length changes most rapidly in proliferative cell populations (e.g., blood). Furthermore, a large Mendelian randomization collaboration (Telomeres Mendelian Randomization et al. 2017) and recent quantitative estimates suggest that both short *and* long telomeres are associated with approximately equal degrees of increased disease risk (Protsenko et al. 2020; Stone et al. 2016). Just as for critically short telomeres, unusually long telomeres are associated with increased risk of tumorigenesis; examples include melanoma and lung cancer (Rode et al. 2016) and women with sporadic and familial breast cancer (Gramatges et al. 2010). An association between longer telomeres and lymphoid cancers has also been demonstrated in individuals with mutations in the ss-telomeric overhang binding shelterin protein POT1 (Ramsay et al. 2013). POT1 mutations associated with long telomeres have recently been reported to confer predisposition to a range of solid neoplasms, as well (DeBoy et al. 2023). Additionally, unusually long telomeres in a cancer-prone family harboring a mutation in the shelterin protein TIN2 that confers telomere elongation with no signs of other defects in telomere function, suggests that long telomeres per se are tumorigenic (Schmutz et al. 2020). These and other contradictory considerations make it clear that a more thorough understanding of how variations in telomere length and sequence affect human health and aging trajectories—*whether on and off the planet*—is necessary.

### The Twin Paradox

The overall goal of the comprehensive and integrated NASA Twins Study was to identify spaceflight-specific factors that influence human health during long-duration missions—important considerations as more and more of us spend longer and longer periods of time, deeper and deeper into space, making our way to the Moon, Mars, and beyond. The Twins Study attracted an astonishing amount of global media attention and excitement, primarily around the fact that the astronaut selected for NASA’s first One Year Mission, Scott Kelly, had an identical twin brother, former astronaut Mark Kelly. Similar in both nature and nurture, the pioneering experiment was conceived: the space twin would spend nearly a year aboard the International Space Station (ISS), while the Earth-bound twin spent that year serving as the genetically-matched ground control (Garrett-Bakelman et al. 2019). The NASA Twins Study represented a number of other important firsts for the U.S. space program, as well. In addition to physiology, cognition, biochemical profiles, microbiome, and immune response studies, it was the first time that NASA ventured into modern “omics” based studies, the gamut of which included genomics (DNA), epigenomics (epigenome), transcriptomics (RNA), proteomics (proteins), and metabolomics (metabolites)—investigations that paved the way for the first DNA sequencing in microgravity with a nanopore instrument (McIntyre et al. 2016), and then on the ISS, performed by astronaut Dr. Kathleen Rubins in 2016 (Castro-Wallace et al. 2017), and eventually the first epigenome data generated during spaceflight (McIntyre et al. 2019).

The Twins Study was also the first time that NASA addressed the question of aging associated with long-duration spaceflight. Indeed, and usually in the context of watching “Interstellar”, Scott was often asked whether he would return from space younger than his brother Mark due to Einstein’s Twin paradox thought experiment in special relativity. However, considering that the ISS is in low Earth orbit (LEO; ~ 250 miles above the Earth) traveling at a mere speed of ~ 17,000 mph, the calculated difference was only ~ a millisecond (0.103 s). Furthermore, upon return to Earth astronauts frequently experience aging-like symptoms, suggesting that spaceflight may actually accelerate the aging process. Thus, the question of aging associated with long-duration spaceflight—and the accompanying risk of developing age-related diseases that could influence performance and survival during a mission, as well as health and aging trajectories afterward—is a critically important one, particularly in the context of space exploration missions, extremely hostile environments and habitation of other planets, and one that we aimed to address directly with our investigation of telomere length dynamics and DNA damage responses in astronauts.

### Telomeres in space

**Fig. 1:**
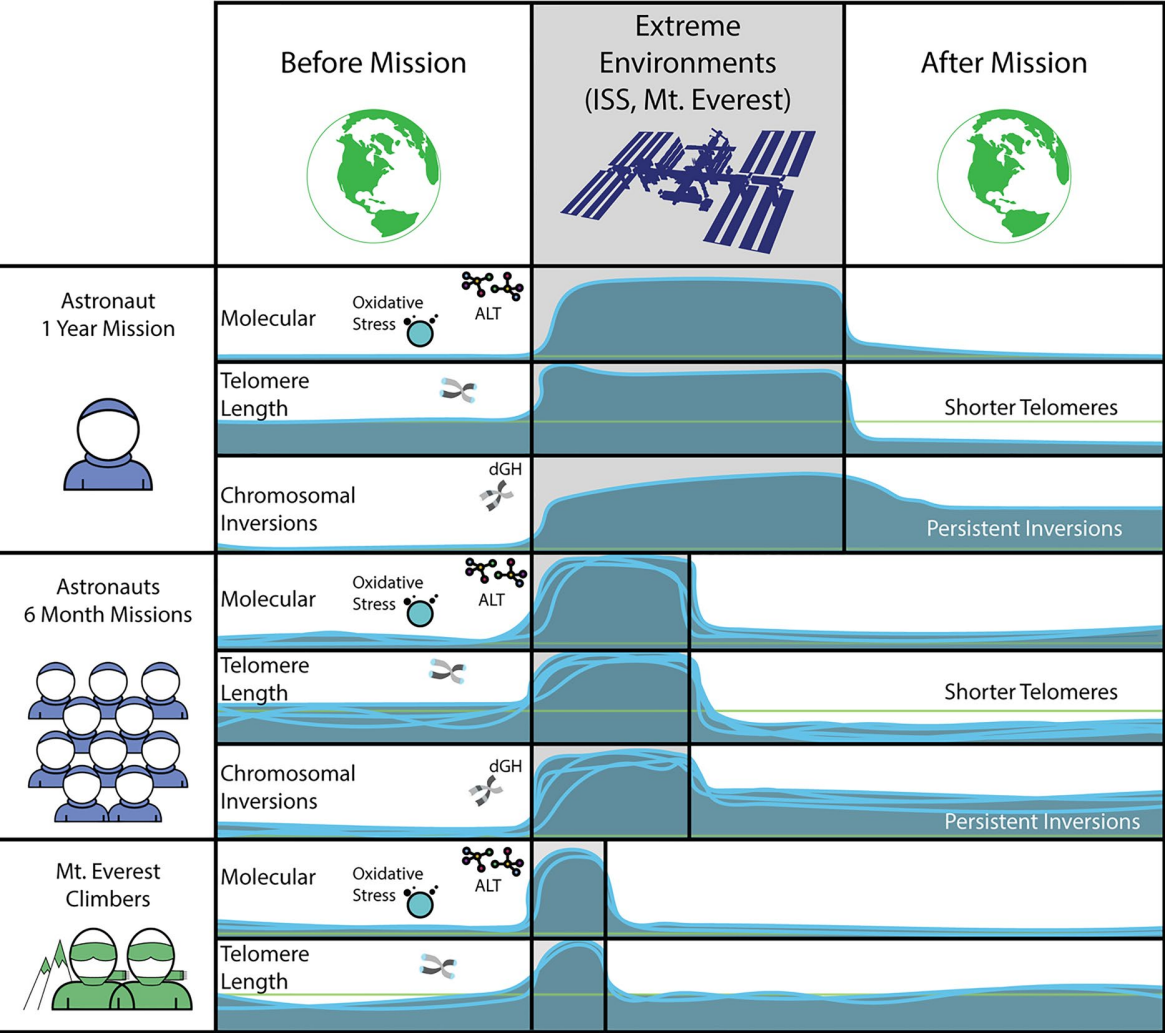
Telomere length dynamics and DNA damage responses before, during, and after 1-year or shorter duration (~ 6 months) ISS missions. Although average telomere length (in blood) was longer during spaceflight irrespective of mission duration, telomere length shortened rapidly upon return to Earth, and overall astronauts had many more short telomeres after spaceflight than they had before. During spaceflight, crewmembers also experienced chronic oxidative stress and evidence of transient activation of the ALT pathway (in normal somatic cells), both of which positively correlated with telomere elongation; similar telomere length dynamics and molecular biomarkers were observed in climbers of Mt. Everest, a somewhat analogous high-altitude extreme environment. Signatures of radiation-induced DNA damage, specifically chromosomal inversions, were significantly elevated during spaceflight and persisted post-flight. Adapted from Luxton et al. (2020a, b)

We documented telomere length dynamics (changes over time) in astronauts experiencing long-duration spaceflight in LEO onboard the ISS (Garrett-Bakelman et al. 2019; Luxton et al. 2020a, 2020b). Similar to our results for NASA’s One Year Mission twin astronaut, which were validated with long-read, single-molecule nanopore sequencing (Grigorev et al. 2021), significantly longer telomeres (in blood) were also observed *during* spaceflight (compared to pre-flight baseline and post-flight measures) in two unrelated 6-month mission astronauts (Fig. 1). Furthermore, and of particular relevance to long-term health outcomes and aging trajectories, telomere length shortened rapidly upon return to Earth and, overall (all astronauts), average telomere length was significantly shorter after spaceflight than before; consistent with this finding, crewmembers also had many more short telomeres after spaceflight than they did before.

Most recently and for the 2021 SpaceX Inspiration4 all civilian crew, telomere elongation (in blood) was observed in all 4 crewmembers during the 3-day, high-elevation (590 km) orbital mission (Al-Turki et al., 2024, *Nature* space biology issue, *Communications Biology, in press*; Overbey et al., 2024, *Nature* space biology issue, *Nature, in press*; Garcia-Medina et al., 2024, *Nature* space biology issue, *NPJ Precision Medicine, in press*). Consistent with our previous studies of astronauts living and working in the space radiation environment (Afshinnekoo et al. 2020), pathways related to oxidative stress, DNA damage responses, and mitochondrial dysregulation were also enriched during the Inspriation4 mission and correlated with telomere length dynamics (Kim et al., 2024, *Nature* space biology issue, *Nature, in press*; Overbey et al., 2024, *Nature* space biology issue, *Nature, in press*), thus supporting this phenotype as a consistent human response to spaceflight independent of mission duration (1 year, 6 months, 3 days). Moreover, our previous cell-by-cell cytogenetic observations of ALT/ALT-like phenotypes *during* spaceflight (heterogeneous telomere lengths) (Luxton et al. 2020b), as well as pathway analyses that implicated ALT and recombination-based maintenance of telomeres in two high altitude climbers (who also experienced telomere elongation associated with ascending Mt. Everest; Fig. 1) (Luxton et al. 2020a), supported our overall supposition that due to telomeres’ particular susceptibility to oxidative damage, the ALT pathway of telomere maintenance may be transiently activated in normal cells during chronic exposure (Luxton et al. 2020b). We have also reported individual differences in response (Luxton et al. 2020a), as well as provided support specifically for the mechanistic role of radiation exposure underlying the changes in telomere length dynamics and persistent DDRs observed (Bailey et al. 2022).

While space flown rodent models (in general) are not appropriate for analogous telomere studies due to their very different than human telomere and telomerase profiles (long telomeres, high telomerase activity, short lifespans), a mutational analysis of *Caenorhabditis elegans* flown on the ISS for 11 days found no significant differences in mutation rates, but did report slightly elongated telomeres in the space-flown worms (Zhao et al. 2006). Moreover, it was recently shown that plants (*Arabidopsis thaliana*) grown onboard the ISS experience highly elevated levels of telomerase activity, which although uncoupled from changes in telomere length (i.e., no significant change), may represent an important redox protective mechanism that promotes survivability in harsh environments (Barcenilla et al. 2023). While there are some ground-based studies that have shown telomere elongation in response to radiation exposure in species as divergent as *Plasmodium falciparum* (Reed et al. 2021), space-flown telomere studies are few, highlighting the importance of better understanding of telomere length dynamics and regulation associated with long-duration spaceflight and chronic exposure to combined stressors, including space radiations, which may provide valuable insight into potential long-term implications for a variety of critical biological systems.

### Space radiation exposure

Space radiation represents a primary hazard and potential “show-stopper” for long-duration space travel and human habitation of other worlds (Afshinnekoo et al. 2020). Particle-based exposures beyond the protection of Earth’s atmosphere include galactic cosmic radiation (GCR; high-energy protons and heavy ions from outside our solar system), solar particle events (SPE; particles shot into space during solar flares), and particles trapped in the Earth’s magnetic field. To date, space-relevant shielding is not practical and other countermeasure strategies have not effectively mitigated the problem of exposure to these very different and damaging ionizing radiations. Appreciation for the regulatory roles of microRNA (miRNA) in controlling gene expression has only recently gained traction in the space community, but holds promise in this regard. Serum miRNAs reliably respond to exogenous stimuli such as ionizing radiation and spaceflight, and so have potential as viable early indicators and biomarkers of exposure. For example, five radiosensitive miRNAs (miR-183-5p, miR-9-3p, miR-200b-5p, miR-342-3p, miR-574-5p) have been identified and a universal model developed to accurately predict exposure to X-ray, ^12^C-ion, and ^56^Fe-ion irradiation (Wei et al. 2017).

Importantly, correct control of gene expression has implications for telomere and genome integrity and stability as well, by way of regulating proteins in the DDR and repair pathways. For example, the ataxia-telangiectasia mutated (ATM) kinase is a miRNA target, with miR-106a and miR-181a capable of upregulating ATM protein levels and decreasing DSBs (Malkani et al. 2020). The ATM pathway is crucial to the DDR network, as ATM acts as a transducer to phosphorylate downstream substrates such as p53, an effector protein and transcription factor. Furthermore, differential expression has been associated with mitochondrial dysfunction; mitochondrial activity is also influenced by ionizing radiation exposure, and it has been found that long-term spaceflight reduces antioxidant defense via reducing mitochondrial enzymes such as superoxide dismutase 2 (SOD2, MnSOD). Such mitochondrial dysfunction has emerged as a nearly universal response observed in spaceflight (Afshinnekoo et al. 2020).

Given their small size, capacity for high-throughput analysis, and cell-free stability in plasma and other fluids, circulating miRNAs have been proposed as functional biodosimeters for space radiation exposure. Malkani et al. (2020) analyzed circulating miRNA signatures in rodent models after exposing them to deep space conditions such as simulated SPE (1 Gy) and GCR (0.5 Gy). They found that the signature was shared in part by human responses ex vivo (irradiated human immune cells) and in vivo (astronaut samples from the NASA Twins Study), suggestive of conservation across species. As for miRNA-based therapeutics, there are two main approaches, these being antisense and replacement therapy. Antisense oligonucleotides (ASOs) inhibit endogenous miRNAs and have been investigated in the context of radiation-induced cardiovascular effects. Reduced angiogenic capacity and loss of vascular integrity was observed in a 3D micro-vessel model exposed to a combination of low- and high-LET particles (Malkani et al. 2020). This effect was attributed to miRNA-mediated bystander transmission and reversed using antagomiRs (synthetic ASOs) to silence upregulated miRNAs associated with the simulated GCR signature (miR-125b-5p, miR-16-5p, let-7a-5p), as determined in (Malkani et al. 2020). Thus, miRNAs and their antagonists may better inform countermeasure and radioprotection strategies. It is intriguing to consider that other RNAs might be expected to do likewise; e.g., telomeric RNA, or TERRA, has recently been shown to play important roles in the radiation response in vivo (Al-Turki *et al.,* 2024, *Nature* Space Biology issue, *Communications Biology, in press*) 10.1038/s42003-024-06014-x.

### Genome editing or gene therapy for future space travelers

Although the vast majority of currently proposed interventions focus on after the fact post-exposure mitigation strategies, it may be possible to augment various defense capabilities pre-exposure or pre-flight. This is the premise of prophylactic genome editing or gene therapy for future space applications. However, and understandably, there exists a gray area between human enhancement and preventative or therapeutic intervention for risk mitigation purposes. From a bioethical standpoint, genetic manipulations to enable space exploration and survival on other planets may be unacceptable in all cases except those in which accrued benefits considerably outweigh risks, for example, if the chance of radiation-induced fatality significantly compromises mission success.

In instances where only one cell type requires editing or modification (e.g., cardiac cells to reduce risk of cardiovascular effects associated with spaceflight), then specificity is paramount. For example, a genome editor intended for the heart could potentially disrupt neurons in the brain or nephrons in the kidney if inappropriately targeted. There are key technical limitations with genome-editing technologies—especially in their delivery. Most base-editing enzymes like CRISPR are too large to fit into adeno-associated virus (AAV) vectors, meaning that other current techniques less commonly used in the clinic must be employed. Another challenge is the precision and control of the editing. If wide-spread temporary change in the expression of a gene is required, potentially augmentable through epigenetic editing, then transient expression of the “epi-editing” machinery may be all that is necessary. However, if a particular gene therapy requires addition of a specific gene into a specific cell that can potentially inappropriately alter the function of other cells, then the editing technology needs to either only embed into the cell type of interest, or at the very least only be expressed within that particular cell type.

Another potentially space-relevant example of cell-type specific in vivo genetic engineering involves the eyes, since vision issues frequently accompany spaceflight. The first CRISPR treatment for a form of blindness called Leber Congenital Amaurosis (LCA) was approved by the FDA in December 2018 for Editas Medicine and Allergan. This new CRISPR therapy was built upon earlier work in gene therapy; e.g., the first AAV LCA therapy (Luxturna) was approved by the FDA in 2017 for treating LCA2, in which the virus carried the payload into the retinal cells to replace the defective gene. Luxturna worked with no known side effects, and a related trial in the Netherlands enabled better vision for 60% of study members. Most recently, the first CRISPR-Cas9 gene editing therapy for sickle cell disease and transfusion-dependent β-thalassemia (Casgevy, or exagamglogene autotemcel) was approved for use in the UK (November 2023). Now with both a viral vector and CRISPR editing, in vivo modification of genes in specific cells make it possible to repair an inherited genetic error.

Telomere-related gene therapies to maintain and/or extend telomere length and thus human lifespan/healthspan are also being developed and tested, and may be of particular relevance to astronauts when they return to Earth and experience accelerated telomere shortening and/or potentially depleted cellular compartments (Luxton et al. 2020a). In 2015, the CEO of BioViva, Elizabeth Parrish, was the first to undergo the company’s anti-aging gene therapy, which included activating telomerase via AAV gene therapy, a highly controversial treatment but prominent example. Telomerase-based gene therapy also has potential for improving treatment of specific age-related degenerative diseases, including Alzheimer’s (e.g., Telocyte). Other approaches and applications include regenerative medicine strategies that employ in vivo delivery of nucleoside-modified TERT mRNA to transiently increase telomerase activity as a means of delaying senescence and keeping young cells young longer (Ramunas et al. 2015). There are also small molecule telomerase activators (e.g., TA-65 developed by Geron) currently available. While potential adverse health effects in the long-term (e.g., increased cancer risk) are currently not known, as such therapies (and others) mature and easy to deliver “anti-aging” drugs prove effective, their use during future long-duration space travel and space exploration has the potential to improve performance and ultimately enable human survival.

Today, clinical trials are run for both somatic and inherited monogenic diseases, including cancer, diabetes mellitus, obesity, hemophilia, age-related macular degeneration, and Huntington’s Disease, and there are a range of over 1000 immunotherapies with genetically modified systems (MacKay et al. 2020). These therapies are also of interest to the space program as a means to confer human radioresistance and reduce risk of disease; NASA and the Translational Research Institute for Space Health are currently funding research to study AAV-based treatments as countermeasures for environmental stressors such as space radiation.

We are indeed witnessing an extraordinary era of therapeutic in vivo genome editing, which is only the beginning. Once the efficacy and safety of such procedures for humans is validated, we will be able to directly edit otherwise fatal or debilitating mutations within an embryo before birth, a genetic engineering approach that could be adapted to success of future space travelers, as well. Deleterious mutations could be closely monitored during development and corrected when necessary to improve the chance of survival and improve quality of life for the individual. And in more futuristic scenarios, desired mutations for improving chances of survival during space exploration and habitation of other planets could also be introduced.

Once these highly complex therapies have demonstrated both efficacy and safety on Earth, we can begin using them in simulated Martian environments, Mars space stations, and even on Mars itself. These types of innovative and unconventional technologies will be crucial to successfully address issues that will most certainly arise when distances from Earth are immense and conditions hostile to human survival, especially given the range of situations in which they can be used, by simply changing a target sequence or delivery system. For example, instead of needing an entire pharmacy where each drug has a different target and use case, a singular system with one modular component (which can be synthesized) can then be adapted to whatever need arises (Nangle et al. 2020).

## Conclusions

Although longer telomeres during spaceflight might be deemed advantageous at first glance, space is likely not a “fountain of youth”, as longer telomere length has also been associated with increased cancer risk (DeBoy et al. 2023). Furthermore, evidence of radiation dose-dependent cell killing was apparent as white blood cell (WBC) counts decreased post spaceflight, consistent with the radiosensitivity of lymphocytes and redistribution of leukocyte subsets during spaceflight (Crucian et al. 2015; Paganetti 2023; Luxton et al. 2021). Overall, astronauts also experienced rapid telomere shortening upon return to Earth and had many more short telomeres after spaceflight than before, the long-term health implications of which could include accelerated aging and increased age-associated disease risk. Thus, space can be viewed as the next frontier of aging studies, breakthroughs in which will be essential for the future success of human space exploration and colonization of other planets, as well as for improving aging trajectories on Earth (Luxton and Bailey 2021), and will also require learning how to survive and adapt to hostile, extreme environments, where distinct gravitational and planetary factors impact every aspect of life. With the ultimate goal of living and thriving in space, a multitude of questions arise, including the most basic, *is longevity in space even possible?*

Currently, reproduction in space—fertility, conception, development, multi-generational effects, and long-term genome stability—are almost all completely unexplored in humans. Therefore, pioneering work will be required in several areas. First, a basic understanding of mammalian embryology in space will be needed for crews to successfully reproduce in space, likely building on murine, primate, and organoid models. While there has only been one married couple in space, and officially no human intercourse in space, a human pregnancy during spaceflight will likely occur in a matter of time as mission durations increase, which has unknown risks for both the embryo and the mother. For example, it will be important to better understand the dramatic shifts in telomere length dynamics that occur during spaceflight, as functional telomeres and telomerase activity are essential for reproduction and embryonic development (Wright et al. 1996). Thus, a second key area of research will be to examine genetic risks in pregnancy and long-term impact on any babies that are born, and who also began gestation while in space. A third key research area will then be to follow crewmembers and their progeny for many years (generations), and compare them to large-scale population cohorts on Earth, to better understand any long-term risk to their genomes, health and aging outcomes. Additionally, advancements in gene therapy technologies will likely be necessary to help ensure proper embryogenesis, development, and successful reproduction on other planets and in the spaceflight environment (Mathyk et al., 2024, *Nature* space biology issue, *npj Women’s Health, in press*).

In the coming years, as more commercial spaceflight providers (e.g., Axiom Space, SpaceX, Sierra Space) and government agencies begin flying longer and longer missions with far more, and more diverse, crewmembers, and as expanded research capabilities in space materialize, future studies will improve statistical power and increase robust conclusions regarding risk. Moreover, once more data becomes available, precision space medicine can inform questions of genome stability, radiosensitivity, and aging trajectories for individual astronauts, and eventually technologies for in situ resource utilization (ISRU) can create the medicines needed during exploration missions (Nangle et al. 2020). For longer-duration and exploration missions, development of effective individualized countermeasures is critical for reducing risk for crewmembers, which may span telomere and telomerase dynamics (general health, disease, aging), as well as space radiation-induced DNA damage responses (e.g., chromosomal inversions; genome instability, cancer), but also metrics for low-frequency mutations like clonal hematopoiesis (Mencia-Trinchant et al. 2020). Taken together, these measures and goals for human research stand to enable humans to become a successful space-faring species, while also better understanding and protecting against genetic, disease and aging risks for themselves and all those who come after, whether on or off our home planet.

## Data Availability

Not applicable.
